# Expression of CMIP in podocytes is restricted to specific classes of lupus nephritis

**DOI:** 10.1371/journal.pone.0207066

**Published:** 2018-11-15

**Authors:** Khedidja Bouachi, Anissa Moktefi, Shao-yu Zhang, Julie Oniszczuk, Kelhia Sendeyo, Philippe Remy, Vincent Audard, Andre Pawlak, Mario Ollero, Djillali Sahali

**Affiliations:** 1 AP-HP (Assistance Publique des Hôpitaux de Paris), Groupe Hospitalier Henri-Mondor, Department of Nephrology and Renal Transplantation, Créteil, France; 2 AP-HP (Assistance Publique des Hôpitaux de Paris), Groupe Hospitalier Henri-Mondor, Department of Pathology, Créteil, France; 3 UPEC (Université Paris Est Créteil), INSERM (Institut National de la Santé et de la Recherche Médicale) U955, Institut Mondor de Recherche Biomédicale (IMRB), Equipe 21, Créteil, France; University of Utah School of Medicine, UNITED STATES

## Abstract

Lupus glomerulopathies are classified into various histological patterns, which probably result from different pathophysiological origins. Podocyte injury can be demonstrated in lupus nephritis but its clinical relevance is far little appreciated and is often masked by proliferative lesions and inflammatory cell infiltrations. Two patterns of podocyte lesions may be considered, either occurring in the context of renal inflammation or reflecting podocyte dysfunction in non-proliferative and non-inflammatory glomerulopathies. This distinction remains elusive since no reliable biomarker discriminates between both entities. CMIP was recently found induced in some glomerular disease but its expression in different lupus nephritis classes has not been investigated. Twenty-four adult patients with lupus nephritis, including non-proliferative (n = 11) and proliferative (n = 13) glomerulopathies were analyzed. Clinical, biological and immunological data were compared with immunomorphological findings. We analyzed by quantitative and qualitative methods the expression of CMIP in different histological classes. We found CMIP abundance selectively increased in podocytes in class II and class V glomerulopathies, while in proliferative forms (class III and class IV), CMIP was rarely detected. CMIP was not expressed in cellular crescents, endothelial cells or mesangial cells. CMIP colocalized with some subsets of B and T cells within glomerular or interstitial mononuclear cell infiltrates but never with macrophages. Hematuria is rarely present in lupus glomerulopathies expressing CMIP. There was no correlation between classical immunological markers and CMIP expression. Thus, CMIP induction in lupus nephritis seems restricted to non-proliferative glomerulopathies and may define a specific pattern of podocyte injury.

## Introduction

Systemic lupus erythematosous (SLE) is a chronic immune complex-mediated disease characterized by a disseminated inflammatory disease, which may affect multiple organs, including the kidney [1]. The autoimmune response involves formation of immune-complexes, which activate the canonical complement pathway, leading to inflammatory lesions and tissue damages, mainly occurring in joints, vessel walls, and kidney, resulting in arthritis, vasculitis and glomerulonephritis, respectively. Lupus glomerulonephritis includes diverse and complex morphological lesions, depending on the proportion of glomeruli affected by active or chronic lesions, the degree of interstitial inflammation or fibrosis, as well as the presence of vascular lesions [2, 3]. Histological evaluation and scoring studies of renal lesions by World Health Organisation (WHO 1982, 1995) have individualized six entities but this classification has evolved over time, because of the diversity of lesions within the same class and the difficulties to ascribe it to clinical or prognostic correlations. Moreover, these classifications fail to optimize the therapeutic strategy, particularly when proliferative lesions are associated with membranous lupus nephropathy. The recent classification from the International Society of Nephrology and Renal Pathology Society (ISN/RPS) distinguishes diffuse glomerulonephritis into separate classes with either segmental (class IV-S) or global (class IV-G) lesions [4]. Although it facilitates clinical study comparisons, this classification fails to improve prediction of disease course.

The pathogenesis processes underlying each type of histological lesion remain unclear [5]. Given the inflammatory nature of proliferative renal lesions, podocyte dysfunction in the context of lupus nephritis is neither clearly individualized nor specifically included in the morphological classification (WHO/INS).

The prevalence of podocyte disease in SLE is not well known, neither its impact on the disease course. Nephrotic syndrome is usually thought to occur in SLE patients in association with immune aggregate deposition on the glomerular capillary wall, frequently accompanied by either endocapillary proliferation or necrosis. However, it can be observed in the absence of immune complex deposits on peripheral capillary walls. Such cases, although uncommon, have been described in association with mesangial lupus nephritis (ISN/RPS, class II), exhibit foot process effacement and are considered as typical podocyte diseases like MCNS or focal glomerulosclerosis [6–8].

*CMIP (Cmaf-inducing protein)* is a recently identified gene that encodes an 86 kDa protein. In physiological situations, *CMIP* is repressed by both WT1 and NF-kB, two major transcription factors in podocytes, which may account for its low levels or non-detection in normal glomeruli [9, 10]. Evidence based *on in vitro* and *in vivo* studies suggest that CMIP induces podocyte signaling disorders and inhibits remodeling of cytoskeleton contributing to podocyte damages [11–15].

In the present work, we aimed to study whether CMIP could be expressed in lupus nephritis, and to determine whether its expression could be correlated with a particular pattern of lupus nephritis.

## Patients and methods

### Patients

All patients analyzed in this study met the criteria for SLE diagnosis based on clinical and laboratory analyses. Kidney biopsy was performed at the time of the onset of renal disease, except in four patients (two class III, one class IV and one class V biopsies). Diagnosis of kidney disease was established by renal biopsy and pathological findings were categorized according to the ISN/RPN classification [4]. Control renal samples were supplied by the hospital tissue bank (platform of biological resources) from patients undergoing nephrectomy for polar kidney tumor. All experiments were conducted with approval from the INSERM (Institut National de la Santé et de la Recherche Médicale) research ethics committee in accordance with international ethics codes and guidelines. Verbal informed consent was obtained from all participants involved in this study.

### Immunohistochemistry analyses

Human kidney biopsies were processed for immunohistochemistry and immunofluorescence studies as described elsewhere [11]. Histological lesions including glomerular injuries, mesangial hypercellularity and tubulointerstitial alterations (mild, +, moderate, ++, and severe, +++) were scored according to the ISN/RPN classification [4]. For quantification, all glomeruli from each section, except those with sclerosis, were analyzed by computer-assisted image analysis using x 400 magnification. Images were blindly analyzed by two independent investigators. Positive staining within each glomerulus was expressed as percentage of immunostained area over total glomerular area using the image analysis software (Image J; National Institute of Health, Bethesda, MD) as previously described [16].

### Laser capture microdissection, reverse transcription and real time quantitative PCR (RT-qPCR)

Because many infiltrating cells express CMIP in some biopsies (classes III and IV), it was not possible to accurately quantify the abundance of CMIP transcript in podocytes on whole tissue biopsies, which required glomeruli isolation by laser capture microdissection. A series of 4 μm-thick sections were cut from frozen renal biopsy specimens using a Leica CM3050 cryostat at -20°C, and mounted onto slides coated with a thermoplastic membrane (Glass PEN-membrane slides; Leica Microsystems, Rueil-Malmaison, France). Normal renal samples were supplied by our Department of Pathology, from patients undergoing nephrectomy for a polar kidney tumor and were considered as normal by pathologists. Cresyl violet staining was performed using a protocol from Zeiss Labs, Munich, Germany [17]. Glomerular structures were selectively dissected using the PALM MicroBeam system (Carl Zeiss). Each kidney biopsy sample was entirely microdissected, giving around 10–20 glomeruli that were dropped into screw-cap vials containing 50 μl of extraction buffer. RNA was extracted from microdissected glomeruli with a picopure RNA isolation kit (Arcturus, Alphelys, Plaisir, France). Reverse transcription (RT) was performed with Maxima First Strand cDNA synthesis Kit (Fermentas).

Real time quantitative polymerase chain reaction (qPCR) was performed using CMIP specific primers (forward: 5’-CGTGTGCCTGGCTGCCATCTACTCCTGCTATG-3’; reverse: 5’-GACAGCGTGGCTTCCTGAGACACCAGGTC-3’). The internal control consisted of PCR amplification of 18S rRNA specific primers (forward: GTAACCCGTTGAACCCCATT; reverse: CCATCCAATCGGTAGTAGCG). Samples (2 μl of the RT reaction mixture, corresponding to 10 ng of equivalent total RNA) were amplified in a 20 μl reaction mixture containing 0.5 mM of each primer and 1 X Quantitect Sybr Green PCR mix (QIAGEN GmbH, Hilden, Germany). Real-time qPCR reactions were performed as recommended by the manufacturer, in triplicate in a Light Cycler 480 apparatus (Roche Diagnostic, Meylan, France). CMIP primers amplified a 176-bp sequence. PCR was initiated by denaturation at 95°C for 15 min, followed by 32 three-step cycles (95°C for 10 s, 68°C for 30 s, and 72°C for 30 s). A dissociation run (95°C for 5 sec followed by 65°C for one min) was performed at the end of PCR program allowing the generation of the melting curve. PCR conditions for 18S rRNA were similar except the annealing temperature (60°C) and the cycle number (n = 20). All PCR data were normalized to 18S rRNA expression, to control for variations in RT reactions. Cycle thresholds greater than 32 were excluded from analyses. Quantification of endogenous CMIP transcript was performed by the 2^ΔΔCt^ method using ribosomal 18S rRNA normalization and results were expressed as -fold induction over values obtained from control samples.

### Statistical analysis

The data presented are means ± SD and were prepared using the GraphPad Prism software, version 6.0 (San Diego, CA). Values of P < 0.05 were considered significant.

## Results

### Characteristics of patients

The population studied includes 24 patients who fulfilled the ARA criteria. Kidney disease was categorized as follow: class II (6 patients); class III (7 patients); class IV (6 patients) and class V (5 patients). The clinical and biological characteristics at the time of biopsy are summarized in Table 1. The mean age at renal biopsy was not significantly different among different groups of patients (P value = 0.859 using one-way ANOVA). The mean interval between SLE diagnosis and onset of lupus nephritis was one year (class II), 1.5 years (class III) and 0.8 years (class IV and class V). Only two patients were male. Among the four groups of patients, striking differences were found in the levels of proteinuria, hematuria, serum albumin, serum creatinine, disease activity index (SLEDAI) and histological activity index (Table 1). On the other hand, there were no significant differences in the levels of serum C3 and C4, ANA titer and serum autoantibodies among groups. However, patients with class III and class IV lupus nephritis had higher levels of anti-dsDNA antibodies. Other autoantibodies including anti-SM, anti-SSA/RO, anti-SSB and anti-PL were detected in some patients.

**Table 1:** Clinical and laboratory findings on patients at the time of biopsy.

Renal Histology	Class II	Class III	Class IV	Class V	P-value (on-way ANOVA)
**Patient Number**	6	7	6	5	
**Age (years)**	31.17 ± 9.86	28 ± 2.82	28 ± 3.45	29,6 ± 3.28	0.8592
**Gender (M/F)**	0/6	0/7	1/5	1/4	
**Proteinuria (g/24 hr)**	2.30 ± 1.56	1.69 ± 1.03	4.76 ± 3.19	7.46 ± 4.12	0.0045
**Hematuria (RBC/ml)**	-	103605 ± 47397	296333 ± 213182	7700 ± 5669	0,0023
SLEDAI*	12.67 ± 6.40	12.86 ± 3.62	18.67 ± 3.72	9.6 ± 1.67	0.0015
**Activity Index**	1	2.86 ± 0.7	8.16 ± 1.81	0.2 ± 0.2	0.0003
**Chronicity Index**	1 ± 0.5	1.5 ± 1	3 ± 2	1 ± 05	0.4360
**Serum Albumin (g/l)**	35.5 ± 11.43	36.79 ± 6.44	19.43 ± 7.57	17.8 ± 4.26	0.0004
**Serum Creatinine (μm/L)**	70.17 ± 8.80	66 ± 10.63	249 ± 243.7	64.2 ± 5.35	0.0004
**GFR-MDRD†** **(ml/min per 1.73m2**	97.83 ± 7.52	97.86 ± 11.6	46.83 ± 37.63	87.4 ± 6.22	0.0007
**Complement C3**	0.36 ± 0.07	0.56 ± 0.30	0.56 ± 0.22	0.64± 0.27	0.2479
**Complement C4**	0.076 ± 0.02	0.088 ± 0.06	0.12 ± 0.10	0.14 ± 0.12	0.5768
**ANA titer^$^**	1/1280 ± 1/640	1/790 ± 1/233	1/1497 ± 1/378	1/688 ± 1/260	0.29
**A-dsDNA positive^¶^**	2	7	6	5	
**A-Sm positive^¶^**	2	-	2	1	
**A-SSA/RO positive^¶^**	4	4	3	4	
**A-PL^§^**	-	-	1	-	

Data are expressed as mean ± SD

* SLEDAI: Systemic Lupus Erythematosus Disease Activity Index

**†** GFR: Glomerular filtration rate

**$** ANA: Antinuclear antibodies

**¶** Number of patients with specific autoantibodies

**§** A-PL: Anti-phospholipid antibodies

Extra renal manifestations include arthritis and skin disease (both classes), Raynaud’s syndrome (one class IV patient), central nervous system vasculitis (one class II patient and two class IV patients), acute pericarditis (one class III patient), cardiac valvular lesions (one class III patient), hematological disorders such as anemia, thrombopenia, (one class III patient and four class IV patients) and antiphospholipid antibody syndrome (one class IV patient). One class IV-patient received steroid (prednisone 10 mg/day) and plaquenil/ Hydroxychloroquine before kidney biopsy.

### Immunohistochemistry analysis of CMIP abundance in lupus nephritis

**Fig 1:**
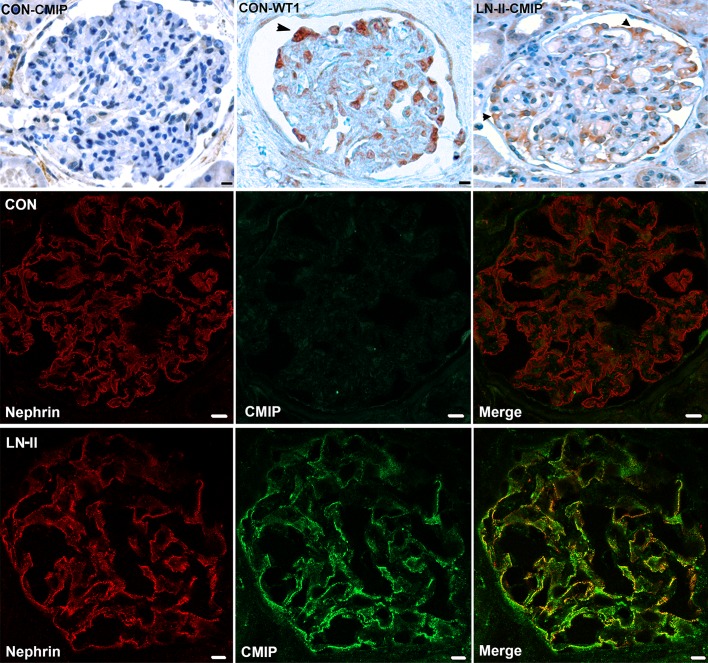
Expression of CMIP in glomeruli from patients with class II lupus nephritis. Representative immunohistochemistry analysis of class II positive biopsies. Top panel: CMIP and WT1 expression in controls (normal human kidney); WT1 labeling shows selective localization in podocytes. Right of the panel: CMIP expression in lupus nephritis class II. CMIP abundance is clearly increased in podocytes with same cellular distribution as WT1, while no detectable expression was seen in tubules. Scale bars, 20 *μ*m. Middle and lower panels: Confocal microscopy analysis of nephrin (red) and CMIP (green) expression in control human kidney (Con) and Class II lupus nephritis (LN-II) biopsies. CMIP abundance is significantly increased in LN-II and colocalizes with nephrin. Scale bars, 10 *μ*m.

***Class II*.** We analyzed 112 glomeruli corresponding to six biopsies (Table 2). In two biopsies totalizing thirty-two glomeruli (patients N°1 and N°2), CMIP was mainly detected in glomeruli, along the external side of peripheral capillary loops, showing similar cellular distribution than WT1, a specific podocyte marker (Fig 1). At the time of biopsy, patient N°1 exhibited nephrotic syndrome, while patient N°2 had significant proteinuria (2.4 gr/24h) and hypoalbuminemia (28 gr/l). The distribution of CMIP was homogenous and appeared restricted to podocytes, in which it was mostly confined to the cytoplasm compartment. Double immunofluorescence labeling showed that CMIP colocalized with nephrin, whereas it was below detection limits in controls (Fig 1, middle and lower panels). No significant signal was detected in tubules or vascular structures. Immunostaining with anti-CD68, anti-CD4 and anti-CD20 did not detect any macrophage or T/B lymphocyte infiltration in glomeruli. In another biopsy with moderate mesangial hypercellularity, CMIP was also expressed in nuclei. No significant signal was detected in tubules or vascular structures. In other biopsies, CMIP expression was scarce or below detection limits.

**Table 2:** Histological and immunohistochemistry findings in class II biopsies.

Patient N°	1	2	3	4	5	6
class	II	II	II	II	II	II
Glomeruli number	I0	22	15	25	20	20
Glomerulosclerosis number	**-**	1	3	1	**-**	**-**
Mesangial hypercellularity	**-**	**-**	**+**	**++**	**++**	**+**
Interstitium alterations	**-**	**-**	**-**	**-**	**-**	**-**
Tubular alteration	**-**	**-**	**-**	**-**	**-**	**-**
Vascular lesions	**-**	**-**	**-**	**-**	**-**	**-**
CMIP labeling	Cytoplasm +++	Cytoplasm +++	Cytoplasm and nucleus	-	-	-

**Fig 2:**
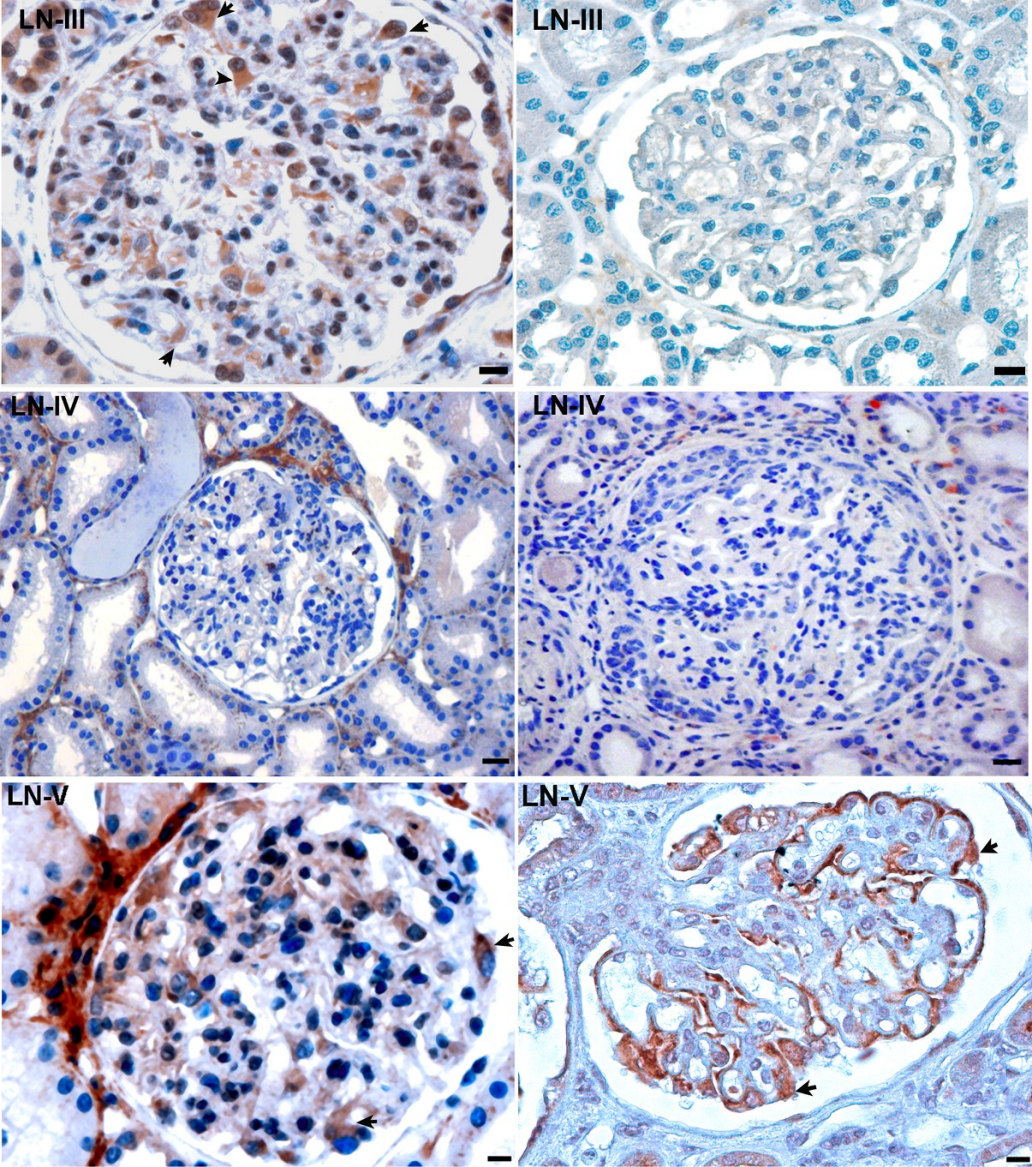
Expression of CMIP in patients with different classes of lupus nephritis. **Upper panel,** expression of CMIP in patients with class III lupus nephritis. In one class III-lupus nephritis (left panel), CMIP is highly detectable in glomeruli with a nuclear and cytoplasmic pattern, while it is not induced in other biopsies (represented by right panel). Scale bars, 20 *μ*m. **Middle panel**, expression of CMIP in patients with class IV lupus nephritis. No significant signal was detected in podocytes, either in cytoplasm or in nucleus compartments. Scale bars, 20 *μ*m. **Lower panel,** expression of CMIP in patients with class V lupus nephritis. Note that CMIP is mostly expressed in the cytoplasm of podocytes. Scale bars, 20 *μ*m.

***Class III*.** We analyzed seven cases totalizing 144 glomeruli (Table 3). In one biopsy (N° 7), CMIP was expressed in both nuclear and cytoplasm compartments of podocytes (Fig 2, upper panel). We did not detect any podocyte labeling in glomeruli of six other biopsies.

**Table 3:** Histological and immunohistochemistry findings in class III biopsies.

Patient N°	1	2	3	4	5	6	7
Class (ISN/RPS)	III-A	III-A	III-A	III-A	III-A	III-A	III-A
Glomeruli number	7	30	21	25	22	26	14
Glomerulosclerosis number	-	-	-	2	-	3	-
Endocapillary proliferation	+	+	+	+	+	+	+
Extracapillary proliferation	-	-	-	+	-	+	
subendothelial deposits	-	-	-	-	-	-	-
Karyorrhexis	-	+	+	-	-	+	-
Fibrinoid necrosis	-	+	+	+	-	-	+
Interstitium infiltration	+	-		-	-	+	-
Tubular lesions	-	-	-	-	-	-	-
Vascular lesions	-	-	-	-	-	-	
CMIP labeling	-	-	-	-	-	-	+ (nucleus)

***Class IV***. We examined 156 glomeruli from six biopsies (Table 4). We did not detect any expression of CMIP in diffuse endocapillary proliferative forms (Fig 2, middle panel). In two biopsies displaying severe extracapillary proliferation, we did not detect any podocyte expression either in cytoplasm or in nucleus compartments. Occasionally, a glomerular signal was observed, which apparently corresponded to infiltrating T and/or B cell expressing CMIP. No signal was detected within the crescents or in the peripheral capillary loops.

**Table 4:** Histological and immunohistochemistry findings in class IV biopsies.

Patient N°	1	2	3	4	5	6
class	IV-GA	IV-GA	IV-GA	IV-GA	IV-GA	IV-GA
Glomeruli number	10	26	20	40	30	30
Glomerulosclerosis number	-	2	-	1	3	-
Endocapillary proliferation	+	-	+	++	+	++
Extracapillary proliferation	+	+++ 14/26	+	-	+++ 21/27	++10/30
Karyorrhexis	-	+	+	+	++	++
Fibrinoid necrosis	-	+	-	-	-	-
Endomembranous deposits and subendothelial deposits (wireloops)	+	+	+	-	++	++
Double contour	-	-	+	+	++	-
Interstitium infiltration	+	+	+	-	+	+
Tubular lesions	-	-	-	-	-	-
Vascular lesions	-	-	-	-	-	-
CMIP labeling	-	-	-	-	-	-

***Class V***. We examined five biopsies totalizing 106 glomeruli (Table 5). The expression of CMIP was restricted to podocytes, mainly in the cytoplasm (Fig 2, lower panel). No signal was detected within the capillary lumen.

**Table 5:** Histological and immunohistochemistry findings in class V biopsies.

Patient N°	1	2	3	4	5
class	V	V	V	V	V
Glomeruli number	16	35	10	15	30
Glomerulosclerosis number	1	-	-	-	-
Mesangial hypercellularity	-	+ 2/35	+ 1/10	-	-
Interstitium aterations	+	-	-	-	-
Tubular atrophy	+	-	-	-	-
Vascular lesions	-	-	-	-	Arterio-sclerosis
CMIP labeling	++ Cytoplasm	++ Cytoplasm	++ Cytoplasm	++ Cytoplasm	+++ Cytoplasm

Collectively, these data suggest that CMIP is strongly induced in podocyte diseases in the context of lupus glomerulopathies but its expression was restricted to non-inflammatory classes such as lupus nephritis classes II and V.

### Immunophenotyping of infiltrating cells

**Fig 3:**
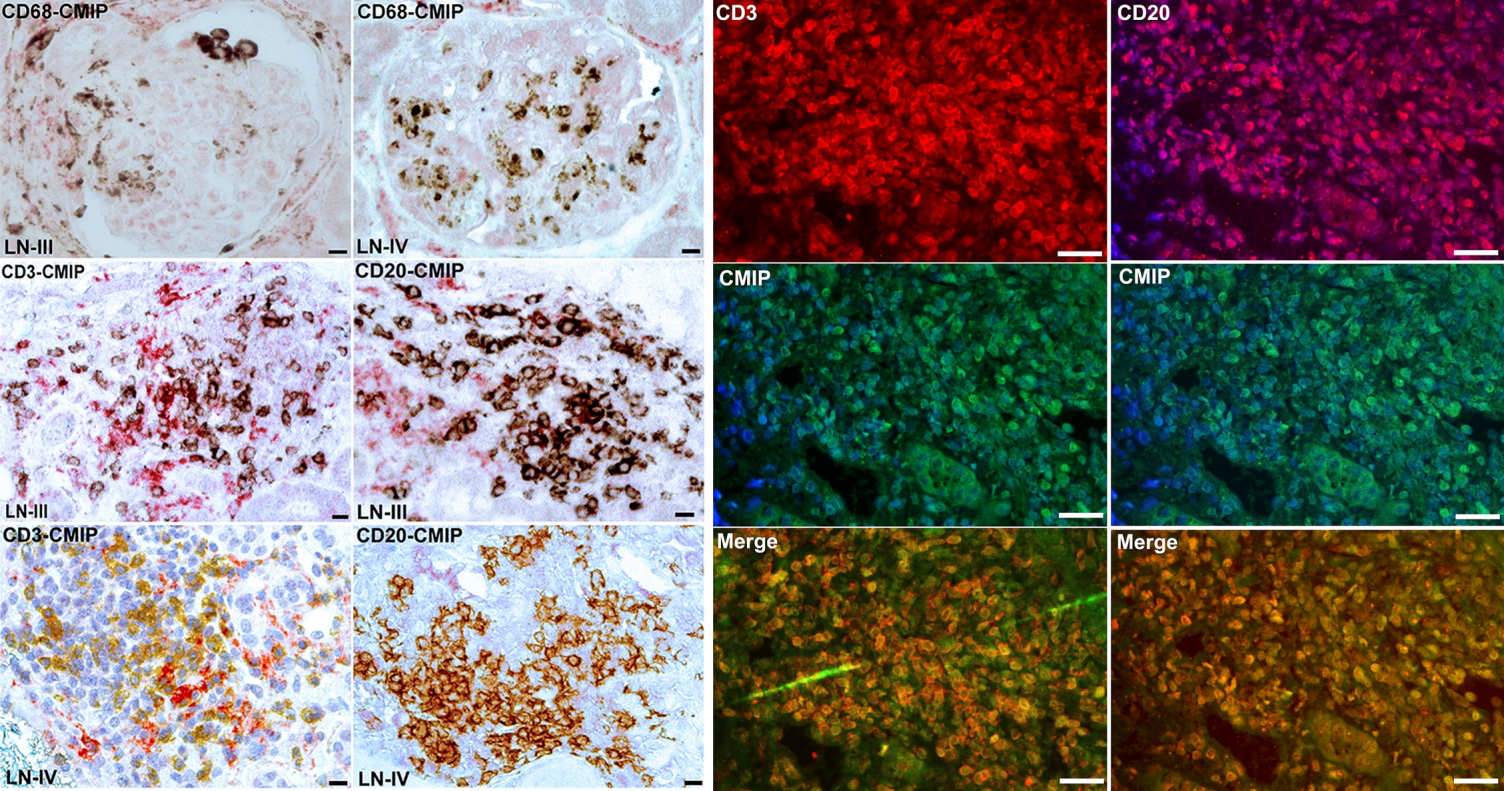
Phenotype characterization of renal infiltrating cells. **Left panel,** Representative immunohistochemistry analysis. Double immunolabeling of kidney biopsies with CMIP (red) and either CD3, CD20 or CD68 (brown) antibodies. Mononuclear cells infiltrating the glomeruli or the interstitium are mainly positive for CD68 (macrophage), CD3 (T cells) and CD20 (B cells) markers. CMIP colocalized with a subset of either CD3 or CD20 positive cells, but never with CD68. Some cells are exclusively positive for CMIP. Scale bars, 20 *μ*m. **Right panel,** immunofluorescence double labeling analysis of kidney biopsies with CMIP (green), CD3 or CD20 (red) antibodies. The colocalisation CMIP and T or B cells is restricted to some lymphocyte subsets. Scale bars, 50 *μ*m.

Classes III and IV lupus nephritis were associated with major mononuclear cell infiltration in interstitial tissues. Double immunolabeling showed that mononuclear cells infiltrating the glomeruli and the interstitial tissues can be differentiated between both types: the infiltrates that were mainly positive for the macrophage marker CD68 did not express CMIP and were prominent in class IV notably within glomeruli, as compared to class III (Fig 3). In the infiltrates expressing CD20 (B cells) or CD3 (T cells), CMIP colocalized with a fraction of these subsets mainly in class III, whereas it was scarcely detected in class IV (Fig 3). These data contrast with the lack of podocyte CMIP expression in classes III and IV and suggest that induction of CMIP in lymphocytes and podocytes relies on distinct pathophysiological mechanisms in SLE.

### Quantification of CMIP in lupus nephritis glomeruli

**Fig 4:**
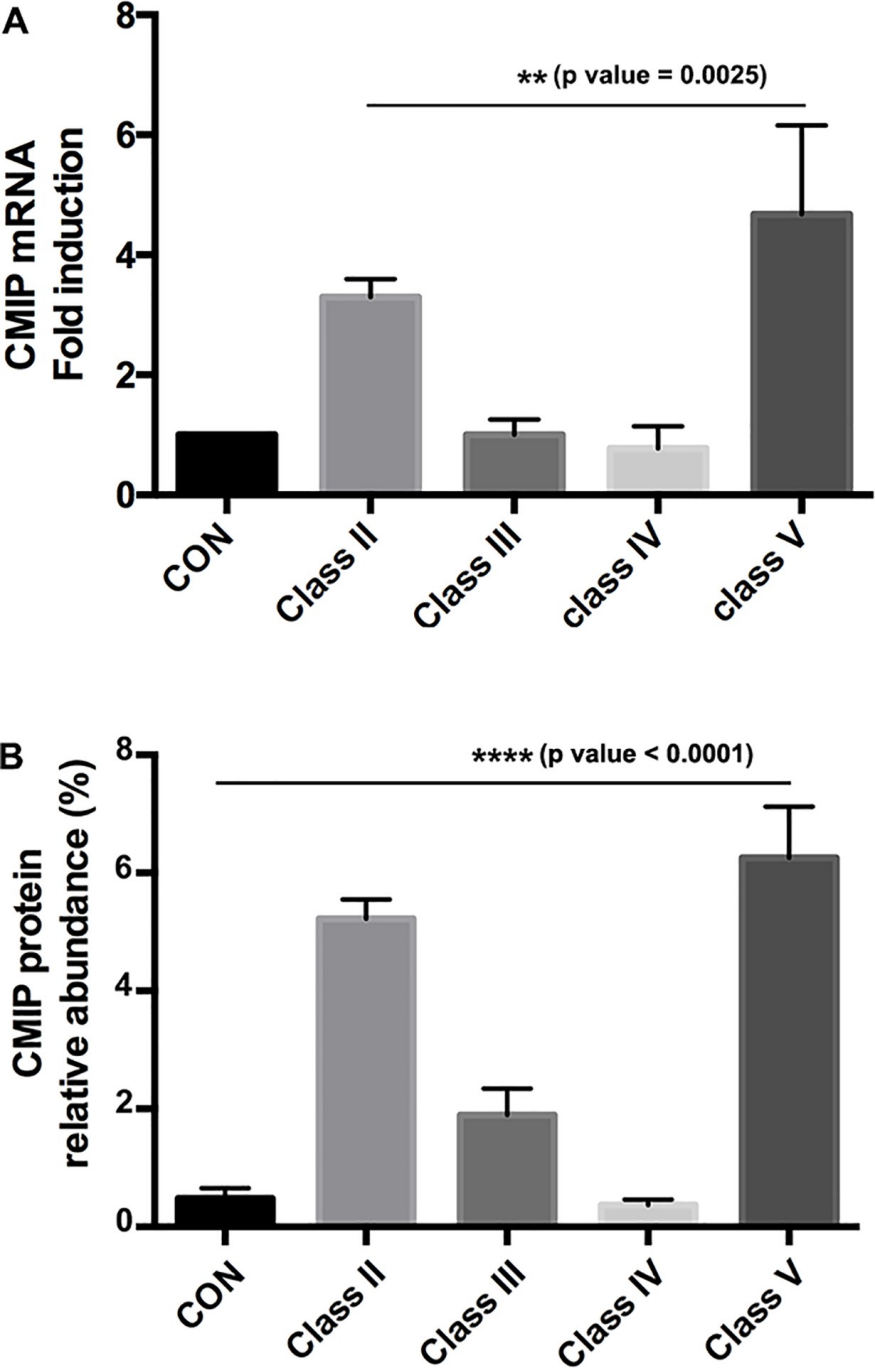
CMIP abundance is significantly increased in non-proliferative lupus glomerulopathies. **A-** Quantitative reverse transcription-PCR of laser-microdissected glomeruli from kidney biopsy specimens (Class II: n = 4 (30 glomeruli); class III: n = 4 (58 glomeruli); class IV: n = 4 (32 glomeruli); Class V: n = 4 biopsies (48 glomeruli) and control kidneys (n = 5 samples, 69 glomeruli). Relative -fold inductions were calculated as described in Methods. Kruskal-Wallis test, **P = 0.0025. **B-** The relative abundance of CMIP was measured by computer-assisted image analysis using X 400 magnification. Positive staining within each glomerulus was expressed as percentage of the immunostained area over total glomerular area using the image analysis software (Image J). Kruskal-Wallis test, ****P< 0.0001.

To assess whether *CMIP* gene was actively transcribed in lupus nephritis and, if so, if its induction was restricted to a particular pattern of glomerular injury, we analyzed the transcript level by quantitative PCR from laser microdissected glomeruli. For each class (II–V) four biopsy specimens were analyzed totalizing 30, 58, 32 and 48 glomeruli, respectively. Quantification of endogenous *CMIP* transcript was expressed as -fold induction over values obtained from control samples. While no apparent difference was observed in classes III/IV relative to controls (Fig 4A), *CMIP* transcript level was significantly increased in classes II/V (Kruskal-Wallis test, **P = 0.0025). Quantification of the relative abundance of CMIP protein was determined for each biopsy in all glomeruli, except in those that were sclerotic. We found that protein expression was significantly increased (Kruskal-Wallis test, ****P*<* 0.0001) and consistent with the transcript level (Fig 4B). These results suggest that CMIP abundance is increased at the transcriptional and protein level in LN-Classes II and V.

### Clinical and histopathological correlations

Hematuria (≥ 10^5^ red cells /ml) was rarely observed in patients who exhibited increased CMIP abundance in podocytes. In classes III and IV, where proliferative and inflammatory lesions are prominent, CMIP was detected neither in podocytes nor in crescents regardless of proteinuria levels. Conversely, in class II and V biopsies, significant proteinuria was correlated with a strong induction of CMIP in podocytes. These data show that CMIP expression is not correlated with proteinuria in lupus nephritis but the variability in the cellular and subcellular distribution suggests that the pathophysiological mechanisms involved in different classes of lupus nephritis are likely distinct.

### Correlation with response to therapy

Three patients with class II biopsy exhibiting high CMIP abundance in podocytes responded promptly to steroid therapy alone (prednisone, 1mg/kg), which was initiated because of extrarenal manifestations including neurolupus (one case) and severe polyarthritis (two cases). In two cases (one class II and one class III) displaying a high nuclear abundance of CMIP in podocytes, the control of renal disease required intravenous cyclophosphamide in addition to steroid therapy with a favorable outcome. The lack of CMIP expression in podocytes of proliferative classes (III/IV) was associated with more aggressive therapy, including high doses of steroids (10/13), mycophenolate mofetil (9/13), cyclophosphamide (5/13) and methotrexate (1/13). Patients with class V biopsy were treated with moderate doses of steroids (prednisone 0,5-1mg/kg) except the patient who later developed a proliferative form (IV GA), requiring cyclophosphamide and Rituximab therapies. These results suggest that CMIP expression in the podocyte does not predict the course of SLE disease.

### Correlation with disease course

One class II patient developed pulmonary hypertension and fatal acute stroke in the context of neurolupus, while kidney disease was in complete remission, four months after the onset of proteinuria. In another class II patient, glomerular disease evolved into class V glomerulonephritis. Among other patients with class II biopsy, three had a favorable outcome, while in one patient (N° 4), kidney disease progressed into class IV-glomerulonephritis within 53 months with a good response to therapy. We did not observe any worse renal course in patients with class III biopsy. In this group, one patient developed polyarthritis, while another presented subacute cutaneous SLE lesions. One patient with class V developed diffuse global proliferative lupus nephritis (class IV GA) within 44 months after the onset of renal disease.

## Discussion

Lupus nephritis occurs in up to 60% of affected adults during the course of their disease. In a recent study, effacement of foot podocyte processes was reported in most lupus nephritis and did not appear to prejudge any class [18]. Two different patterns of podocyte injury based on distinct pathological mechanisms are clearly suggested, but there is no reliable biomarker discriminating both entities [8]. In non-proliferative lupus glomerulopathies, proteinuria may occur with (class-V) or without (class-I) evidence of immune complex deposits in glomeruli. In this study, we provide evidence that: i) CMIP is selectively expressed in the cytoplasm compartment of podocytes in class II and class V glomerulopathies, suggesting that podocyte is presumably the main target in these lupus glomerulopathies; ii) in proliferative forms (class III/IV), CMIP is almost not expressed either in podocytes or in cellular crescents; iii) there is no correlation between classical immunological markers and CMIP expression; iv) expression of CMIP in podocytes is associated with a better response to therapy. However, one patient with class II lupus nephritis and overexpressing CMIP in podocytes died of active neurolupus.

The participation of podocyte in crescent formation remains a matter of debate. Early studies have found that most cells forming crescents do not express any podocyte markers, including CD10, podocalyxin, synaptopodin, glomerular epithelial protein 1, podocin, CD2AP, nephrin, and Wilms tumor antigen I (WT1), suggesting that podocytes are not involved in the formation of crescents [19, 20]. This concept has been challenged by recent identification of new podocyte markers, including nestin, Hip1 and Olfml2, which have been found expressed by crescent cells, regardless of initial injury [21, 22]. It is noteworthy that the expression of these markers contrasts with the loss of usual mature podocyte markers such as podocin, nephrin and WT1. Our data showing that CMIP is not expressed in crescents, while it is induced in non-proliferative classes, suggest a de-differentiation of podocytes in crescent forming areas.

Recent studies have shown that parietal epithelial cells (PEC) predominate in crescents, based on the higher cell positivity for cytokeratin-8. Although the cell origin of crescent-forming cells is difficult to establish, animal models provide an experimental cue showing that selective PEC depletion induces remaining PECs to proliferate and form crescent [23].

Renal progenitor cells are a subset of PEC defined by co-expression of CD24 and CD133 stem cell markers, which potentially give rise to tubular and podocyte cells [24]. In crescentic glomerulonephritis, CD24^+^ CD133^+^ proliferating cells constitute the major contingent of the hyperplastic lesions whereas podocytes included in these lesions do not proliferate [25]. Given the inflammatory nature of crescents, one might postulate that some mediators released in the microenvironment of crescent deregulate the genetic program of differentiated podocytes, leading to a loss of classical markers. This deregulation process may trigger the expression of neo PEC antigens in podocytes.

CMIP expression was only detected in three out of six patients with class II biopsy, who remained free of relapse during the follow-up. One CMIP-negative-class II patient developed a more aggressive course (class IV). In a series analyzing the course of nineteen patients with class II biopsy, only eight patients achieved a complete remission, while nine patients were nonresponders and developed a higher-grade nephritis [26]. Altogether, these observations highlight the heterogeneous nature of this class, which possibly involves different pathophysiological mechanisms. It would be interesting to study whether CMIP expression in class II could differentiate the two course profiles.

We have recently shown that CMIP prevents translocation of RelA to the nuclear compartment, which results in inhibition of NF-κB activation [27]. We have also reported that CMIP interacts with Dip1 and upregulates DAP kinase, which has been found to inhibit NF-κB activation [28, 29]. Inhibition of NF-κB activity is independent of cell line since it has been observed as well in cells of hematopoietic origin as in podocytes [12, 27]. These results suggest that CMIP might interfere with NF-κB activity by several mechanisms and raise the possibility that CMIP induction prevents the release of proinflammatory mediators by the podocyte and contribute to reducing the recruitment of inflammatory cells within the glomeruli. Conversely, the lack of podocyte CMIP expression in proliferative forms (class III and IV) might result from factors released by infiltrating leukocytes or proliferating mesangial cells, which block transcription of *CMIP* gene in podocytes. We postulate that in these proliferative forms the release of proinflammatory mediators including tumor necrosis factor-α (TNF-α), interleukin-1ß (IL-1ß) and interleukin-6 (IL-6) may induce NF-κB activation in podocytes, which, in return, blocks transactivation of *CMIP* gene [9].

## Conclusion

We report for the first time the expression of CMIP in lupus nephritis. Our results suggest that induction of CMIP in lupus nephritis appears to be limited to non-proliferative forms and may define a specific pattern of podocyte injury. We did not find any evident correlation between classical immunological markers and CMIP expression. However, the latter is associated with a better renal course of the disease.
